# The stable cyclic adenosine monophosphate analogue, dibutyryl cyclo-adenosine monophosphate (bucladesine), is active in a model of acute skin inflammation

**DOI:** 10.1007/s00403-012-1216-6

**Published:** 2012-02-03

**Authors:** Chris Rundfeldt, Hartwig Steckel, Torben Sörensen, Piotr Wlaź

**Affiliations:** 1 Drug-Consult.Net, Toepfferspark 2a, 39108 Magdeburg, Germany; 2 Department of Pharmaceutics and Biopharmaceutics, Christian Albrecht University Kiel, Grasweg 9a, 24118 Kiel, Germany; 3 Department of Animal Physiology, Institute of Biology and Biochemistry, Maria Curie-Skłodowska University, Akademicka 19, PL-20033 Lublin, Poland

**Keywords:** Dibutyryl cyclo-adenosine monophosphate, Inflammation, Anti-inflammatory, Arachidonic acid, Mice

## Abstract

Anti-inflammatory therapeutic options for the topical treatment of skin diseases with inflammatory or allergic contribution are mostly limited to topical glucocorticoids and calcineurin inhibitors. Both compound classes induce adverse effects. Elevation of intracellular cyclic adenosine monophosphate (cAMP) by inhibition of phosphodiesterase 4 was shown to induce potent anti-inflammatory effects, but the safety profile of currently available compounds is not sufficient. A different approach to increase intracellular cAMP is the substitution of chemically stabilized cAMP analogues. Bucladesine is a stabilized cAMP analogue with an excellent safety profile which had been marketed as topical treatment of impaired wound healing. In the current study, a novel water free emulsion containing bucladesine was evaluated for anti-inflammatory effects. In the arachidonic acid induced ear oedema model in mice, single or multiple administration of an emulsion containing 1.5% was capable of significantly reducing the inflammatory oedema. The data indicate that bucladesine represents an interesting treatment option for skin diseases where an anti-inflammatory activity is indicated. Due to the established clinical safety, this agent may bridge the gap between potent agents such as glucocorticoids or calcineurin inhibitors and emollients without active compounds.

## Introduction

Cyclic adenosine monophosphate (cAMP) plays an important regulatory role in virtually all cell types involved in the pathophysiology of allergic and inflammatory diseases. Phosphodiesterase 4 (PDE4) is the major cAMP degrading isoenzyme in inflammatory and immune cells and in keratinocytes. Inhibition of PDE4 results in broad anti-inflammatory/immunomodulatory action which can be related to cAMP elevation [15, 19]. PDE4 inhibitors are active in models of non-immunological skin inflammations such as the arachidonic acid induced skin inflammation in mice [4], in models of allergic contact dermatitis in mice, and in ovalbumin sensitized guinea pigs [14]. Results of early clinical trials with both topical treatment with cipamfylline [10] and systemic treatment with atizoram (CP-80,633) [12] or apremilast [8] demonstrated efficacy of PDE4 inhibitors in atopic dermatitis and psoriasis.

The utility of PDE4 inhibitors is limited by the safety profile and drug-induced gastrointestinal adverse effects such as nausea or emesis likely represent class effect toxicity [13]. Due to this toxicity, the maximum tolerated dose is either sub-therapeutic or at the very bottom of the efficacy dose–response curve [7]. Roflumilast is currently the only selective PDE4 inhibitor marketed for the treatment of chronic obstructive pulmonary disease, but has a narrow safety window [18].

N^6^,2-O-dibutyryladenosine-3′,5′-cyclic monophosphate (bucladesine, DB-cAMP) is a membrane permeable stabilized analogue of the endogenous mediator cAMP. Bucladesine is more lipophilic than cAMP and in contrast to cAMP capable of penetrating cell membranes [3]. It is not cleaved by PDE but instead acts as an enzyme blocker. In addition bucladesine interferes with different protein kinases which are normally activated by cAMP [16]. Bucladesine has undergone in the past clinical developments as systemic treatment for cardioprotection and as topical treatment to improve wound healing. In Japan, a bucladesine ointment (Actosin® ointment; Daiichi Pharmaceutical Co., Ltd., Tokyo, Japan) was marketed to treat skin ulcers. Clinical studies have shown favourable effects on diabetic foot ulcers or decubitus, but the compound was later withdrawn despite good tolerability [1, 2]. One possible reason for the withdrawal may be the odour of the cream formulation which can be related to the hydrolytic cleavage in aqueous solutions resulting in release of butyric acid [17].

The aim of this study was to evaluate whether bucladesine may be capable of suppressing non-immunological skin inflammation, utilizing the model of arachidonic acid induced acute inflammation. The experiments aim at profiling bucladesine as potential new treatment for inflammatory or allergic skin diseases. A further aim was to evaluate the in vivo activity of a novel water free ointment, developed to prevent hydrolysis and thus preventing generation of butyric acid.

## Materials and methods

### Development of a topical cream formulation

Bucladesine is readily water soluble, but is not stable in aqueous solutions. At room temperature about 20% of an aqueous solution of bucladesine is hydrolyzed within 8 days and the speed of hydrolysis is further increased at pH values below 5 and above 7 (data not shown). It was our aim to find a water free formulation where bucladesine is presented in solution to prevent inhomogeneity and instability resulting from crystal growth or chemical degradation. To find adequate solvents, a screen was conducted [Sörensen T (2007) Stabilization of cyclic AMP derivatives in pharmaceutical preparations for topical application. Diploma Thesis, Department of Pharmaceutics and Biopharmaceutics, Christian Albrecht University, Kiel, Germany]. The solubility of bucladesine was determined in different solvents, using a semi-quantitative approach. To estimate solubility, 8–10 mg of compound was dissolved in 1 g of different solvents by vigorous shaking for 1 h and visual control of solubilisation. If complete solubilisation was achieved, the saturation concentration was estimated by adding step-by-step further small quantities of bucladesine until crystalline residues became visible in the vial. Bucladesine is not well soluble in hydrophobic ingredients such as silicones, triglycerides or hydrocarbons, but it is well soluble in propylene glycol, ethylene glycol, and *n*-methyl pyrrolidone (Table 1). Silicone fluid 1250 was selected as outer phase for an emulsion, while *n*-methyl pyrrolidone was selected as inner phase, capable of carrying about 10% of bucladesine. Bucladesine was found to be stable if dissolved in *n*-methyl pyrrolidone; no hydrolysis was observed within 14 days (data not shown). Long term stability was not conducted for the purpose of this experimental study. The silicone emulsifier Abil Care 85® (Evonik Goldschmidt GmbH, Essen, Germany) was selected and, if used at a concentration of 2%, resulted in a stable emulsion if prepared at lab scale using the IKA laboratory reactor IKA-LR 250 (IKA Werke GmbH & Co. KG, Staufen, Germany). The emulsion was visibly stable for a period of >14 days (maximum observation period) and no sign of hydrolysis, such as increased butyric acid odour, was observed. The emulsion was also shown to be stable if tested using a centrifuge assay at up to 5000 rpm for 3 min [11]. Two different emulsions were selected and prepared for animal testing. The 0.5% emulsion contained 88% silicone fluid 1250 and 10% *n*-methyl pyrrolidone containing 50 mg/g bucladesine, stabilized with 2% Abil Care 85®. The high concentration emulsion contained 83% silicone fluid 12500 and 15% *n*-methyl pyrrolidone containing 100 mg/g bucladesine, stabilized with 2% Abil Care 85® resulting in a cream formulation containing 1.5% bucladesine.

**Table 1:** Solubility of bucladesine in different solvents

Solvent	Supplier	Solubility (mg/g)
Paraffin oil	Evonik Goldschmidt GmbH, Essen, Germany	<8
Oleyl alcohol	Evonik Goldschmidt GmbH, Essen, Germany	<8
Miglyol® 812	Caesar & Loretz GmbH, Hilden, Germany	<8
Labrafac Lipo WL 1349	Gattefossé GmbH, Weil am Rhein, Germany	<8
Silicone fluid Q7-9120, 20 cSt	Dow Corning, Wiesbaden, Germany	<9
Silicone fluid Q7-9120, 12500 cSt	Dow Corning, Wiesbaden, Germany	<10
*n*-Methyl pyrrolidone	International Speciality Products, Wayne, NJ, USA	>112
Propylene glycol	Sigma-Aldrich, Munich, Germany	>114
Ethylene glycol	Sigma-Aldrich, Munich, Germany	>151

As reference solution, bucladesine was dissolved immediately prior to the experiment in a mixture of 100 μl DMSO and 900 μl water at a final concentration of 5%.

### Arachidonic acid induced ear swelling as a model of non-immunological inflammation

Experimentally naïve male Albino Swiss mice (Laboratory Animals Breeding, Słaboszów, Poland) weighing 20–30 g were used in all experiments. The animals were housed in polycarbonate cages at a controlled temperature (23–25°C) and humidity (50–60%) with 12 h light/dark cycle (lights on at 6 h). Tap water and food pellets were available ad libitum. All experiments were performed after at least 7 days of acclimatization. The experimental protocols were approved by the Ethical Committee of the Medical University in Lublin (license number 40/2010).

The anti-inflammatory effect of bucladesine was compared to the respective vehicles and the reference drug ketoprofen gel 2.5% (Fastum Gel, A. Menarini, Florence, Italy).

For topical administration of bucladesine as 5% solution, 20 μl of drug or vehicle solution was administered onto the outer surface of both, left and right ears each, 60 min prior to arachidonic acid challenge. The inflammatory response was induced by administration of 20 μl arachidonic acid (Sigma-Aldrich, Munich, Germany; 5% in acetone) on the outer surface of left ears. The right ears were treated with acetone only to determine the individual differences in ear thicknesses.

To allow for skin penetration of the cream formulations and based on preliminary experiments with ketoprofen cream in this model (data not presented), a pre-treatment time of 3 h was selected. To evaluate the effect of 0.5 or 1.5% bucladesine cream, an amount of 10 ± 1 mg cream each was spread onto the outer surface of both, left and right ears using small spatula. A third group of mice was administered with 2.5% ketoprofen gel. Corresponding vehicle treatment groups were administered either the respective cream base without bucladesine or, as reference for ketoprofen, a cosmetic night cream. To evaluate the effect of repeated administration, four separate groups of mice were dosed twice, i.e., 7 and 3 h before administration of arachidonic acid with the 0.5 and 1.5% cream or corresponding vehicle. Arachidonic acid or actetone were administered 3 h after the second cream administration onto left and right ears, respectively. The ear thickness was measured with a precise spring-loaded caliper (Mitutoyo, type 7309, Kawasaki, Japan) before and 60 min after application of arachidonic acid or vehicle.

### Statistical analyses

The increase in ear thickness was calculated for each mouse as the difference between left ear thickness before and 60 min after administration of arachidonic acid. To account for individual variability the difference between the right ear thickness before and 60 min after administration of acetone was subtracted from the arachidonic acid induced increase in left ear thickness. Mean and standard deviation (SD) was calculated from single ear oedema values. One-way analysis of variance (ANOVA) with Dunnett’s post hoc test or Student’s *t* test, where appropriate was applied.

## Results

**Fig. 1:**
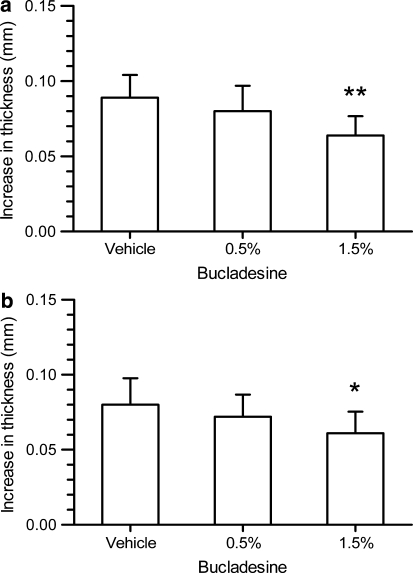
Anti-inflammatory effect of 0.5 and 1.5% bucladesine cream given **(a)** 3 h before administration of arachidonic acid [one-way ANOVA: *F*(2,27) = 7.062; *P* = 0.003] or given **(b)** twice, i.e., 7 and 3 h before administration of arachidonic acid [one-way ANOVA: *F*(2,27) = 3.695; *P* = 0.0382]. *P < 0.05, **P < 0.01 versus vehicle-treated mice (one-way ANOVA with Dunnett’s post hoc test)

Bucladesine, administered topically as water-free cream formulation, was capable of reducing the arachidonic acid induced ear swelling. If administered as 0.5% water-free emulsion, a 10% reduction in ear swelling was reached (not statistically significant). The effect was increased if the concentration of bucladesine was increased to 1.5%, resulting in a 28% reduction in ear swelling (*P* < 0.01%, Fig. 1a).

Twice administration of the cream resulted in an effect which was nearly identical to the single administration, i.e., 10% reduction at 0.5% and 24% reduction at 1.5% bucladesine, the later effect being statistically significant (*P* < 0.05, Fig. 1b).

**Fig. 2:**
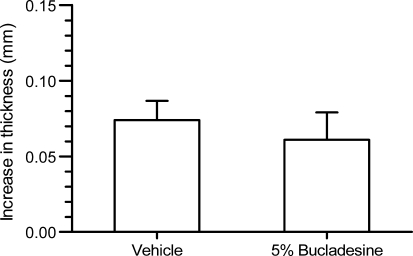
Anti-inflammatory effect of 5% bucladesine given 1 h before administration of arachidonic acid

The administration of bucladesine as 5% solution in DMSO:water only resulted in a modest (18%) reduction in ear swelling and this effect failed to reach level of significance (*P* = 0.071, Fig. 2).

**Fig. 3:**
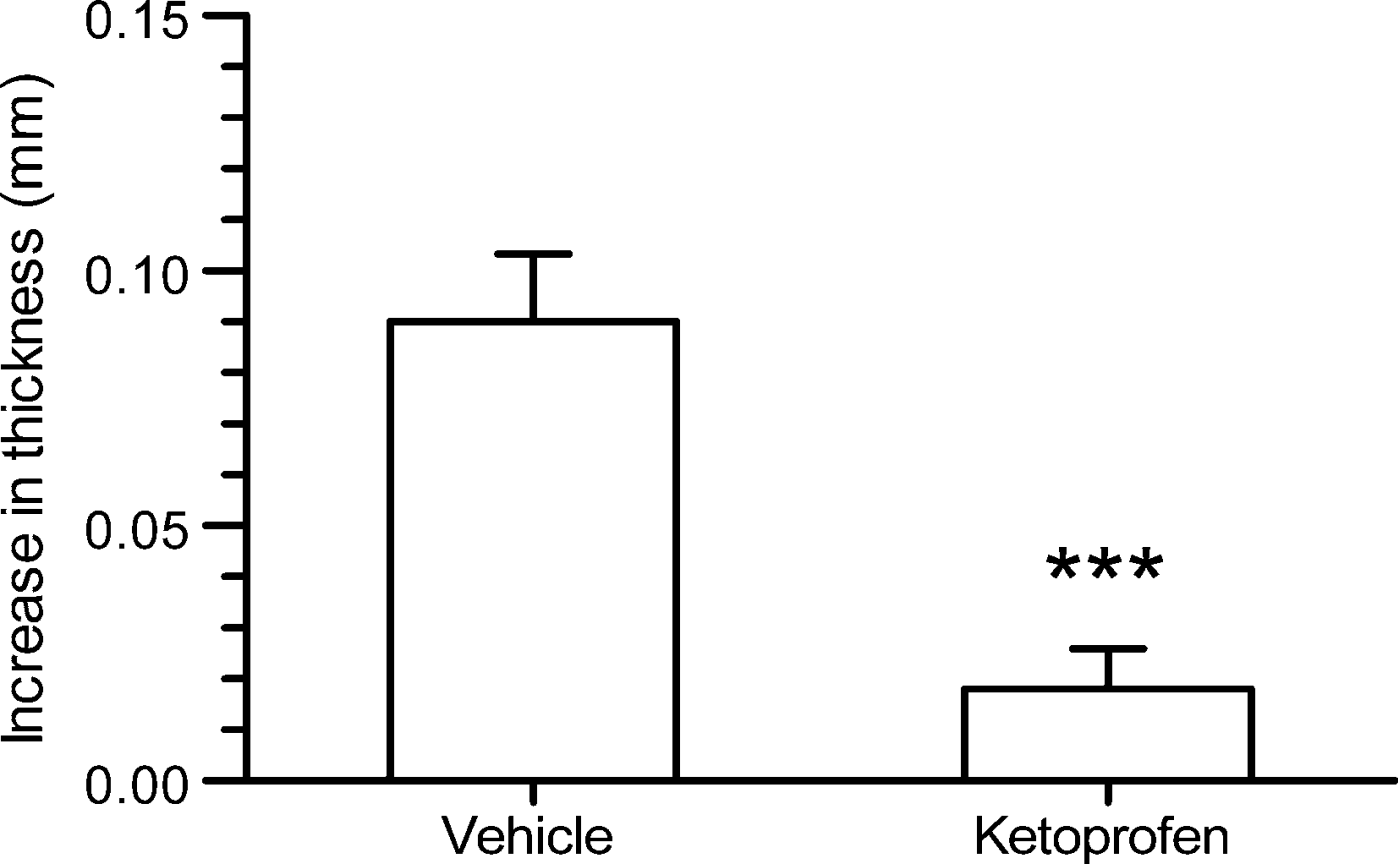
Anti-inflammatory effect of 2.5% ketoprofen gel given 3 h before administration of arachidonic acid. ***P < 0.001 versus vehicle-treated mice (Student’s *t* test)

As expected, 2.5% ketoprofen gel nearly completely suppressed the arachidonic acid induced ear swelling (Fig. 3).

## Discussion

Minimal anti-inflammatory treatment of the underlying residual skin disease by prophylactic application of immune modulating agents is discussed as an option to prevent the outbreak of acute symptoms in well-controlled patients [20]. However, therapeutic options are mostly limited to topical glucocorticoids and calcineurin inhibitors, two compound classes with tolerability issues especially in children [6]. While PDE4 enzyme inhibition may be an interesting immune modulatory option, the safety profile of currently known inhibitors is not optimal [5]. Even the topical administration of the weak PDE4 inhibitor cipamfylline induced gastrointestinal adverse effects, but was at the same time shown to be pharmacologically active in patients with atopic dermatitis [10].

An alternative approach to elevate the intracellular cAMP level is the topical application of a stable analogue of cAMP. Bucladesine is well tolerated and no PDE4-like adverse effects are known from its clinical use as cream formulation [2].

We now show that bucladesine, if administered as water-free topical cream formulation, is active in an animal model of acute skin inflammation. The activity of bucladesine in this model is comparable in size to the activity of the highly potent PDE4 inhibitor AWD 12–281 in the same model, if administered at a concentration of 0.3–1%. At higher concentrations (3 and 5% ointment), AWD 12–281 was nearly equipotent to indomethacin, indicating that selective PDE4 inhibitors are more potent than bucladesine [14]. If administered as 5% solution in DMSO/water, the reduction in ear oedema did not reach level of significance. These data indicate that an adequate cream formulation is required to enable the pharmacological effect of bucladesine. No potentiation of the activity was achieved if the compound was administered twice, indicating that the effect is of short duration.

While in the current study, the arachidonic acid model was utilized as a simple model of pharmacological activity, other models of skin inflammations involving Th1- and Th2-dominated immunological reactions have been shown to be sensitive to elevation of intracellular cAMP [5]. Therefore, this treatment strategy may indeed be applicable for the topical treatment of a wide variety of skin diseases. Indeed, clinical data indicate that elevation of cAMP as therapeutic principle was active in a human model of acute skin inflammation, the balm of Peru induced skin irritation [9].

In conclusion, bucladesine represents an interesting treatment option for skin diseases, where an anti-inflammatory activity is indicated. Due to the established clinical safety, this agent may bridge the gap between potent therapeutics such as glucocorticoids or calcineurin inhibitors and emollients without active compounds.

However the odour of butyric acid represents a challenge. Despite the fact that the currently developed formulation was found to be stable within the scope of this investigation, even minimal amounts of butyric acid are barely tolerable. In future studies, the activity and safety of a different analogue of cAMP, namely 2-deoxy-cAMP should be evaluated since it can be expected to have a very similar pharmacological profile, without the risk of butyric acid generation.
